# Recurrence of melanoma in the scar after excised Spitz nevus in a 17-year-old child^☆^^☆☆^

**DOI:** 10.1016/j.abd.2020.07.014

**Published:** 2021-05-21

**Authors:** Joanna Pogorzelska-Dyrbuś, Beata Bergler-Czop, Maciej Kajor

**Affiliations:** a “Estevita” Specialist Medical Practice, Tychy, Poland; b Department of Dermatology, Medical University of Silesia, Katowice Francuska, Poland; c Department of Pathomorphology and Molecular Diagnostics, School of Medicine in Katowice, Medical University of Silesia, Katowice, Poland

**Keywords:** Melanoma, Nevus, epithelioid and spindle cell, Recurrence

## Abstract

Melanoma in childhood is rare and its diagnosis is more difficult than in adults, as it often presents histologic features overlapping with the Spitz nevus. The authors report the case of a 17-year old boy who was first diagnosed with Spitz nevus, however, the final diagnosis made after the excision of the tumor arising in the scar was changed to melanoma. The case in this present study emphasizes the importance of the differential diagnosis of skin tumors in young patients.

## Introduction

Spitz nevi are benign tumors usually arising during childhood as rapidly growing non-pigmented -pink or red papules or nodules most often of the lower extremities or on the face.^1^ The authors report the case of a 17-year-old boy who was first diagnosed with spitz nevus, but the final diagnosis made after the re-excision of the scar was changed to melanoma.

## Case report

A 17-year-old boy presented to the Specialist Medical Practice in Tychy, Poland, with a tumor increasing in the scar on the left thigh six months after surgical excision of a nevus in this site. The excised, pink 5-mm rapidly growing tumor had been subjected to histopathological examination and Spitz nevus had been diagnosed. The patient denied preceding trauma and a family history of melanoma. There had been no dermoscopy before the surgical procedure.

**Figure 1:**
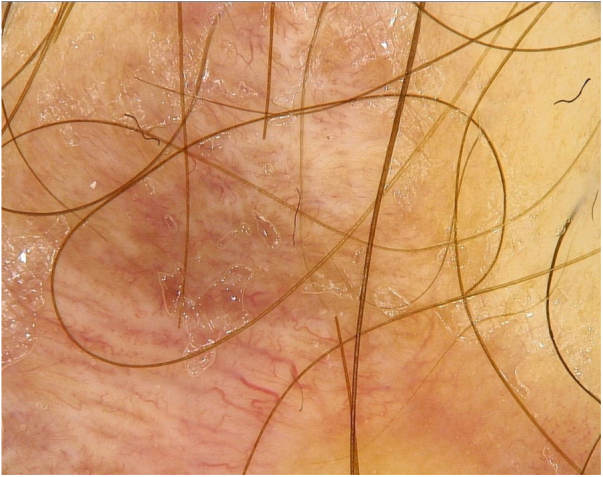
The dermoscopic image of the first scar. The polymorphic pattern with a predominance of linear irregular vessels was visible.

Clinical examination of the red scar revealed the presence of the shining nodule in its lower segment. In dermoscopy, the polymorphic pattern was present with a predominance of linear irregular vessels (Fig. 1). Considering dermoscopic features and the diagnosis of Spitz nevus, the patient underwent subsequent excision of the scar and histopathological diagnosis of recurrent Spitz nevus was established.

**Figure 2:**
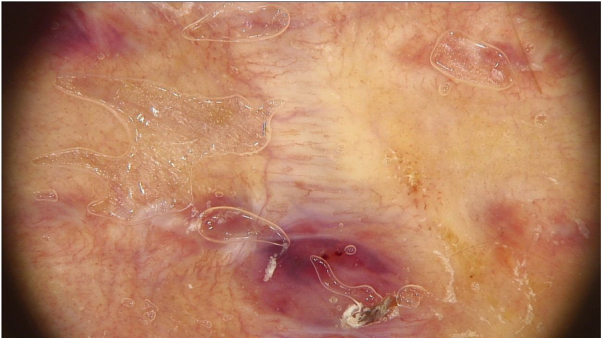
The dermoscopic image of the second scar. The polymorphous vascular pattern consisted of dotted and linear vessels along with a “milky red area”.

**Figure 3:**
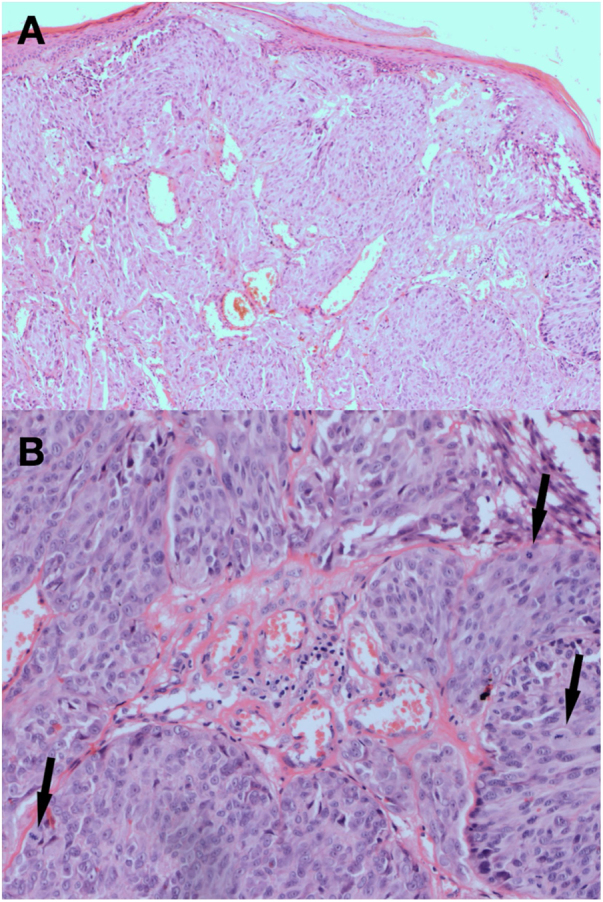
Histopathological image of the melanoma. Caption: Nodular melanoma, epithelioid type, Clark 3, Breslow 2.39-mm. (A), Hematoxylin & eosin ×40, (B), Hematoxylin & eosin ×100. The arrows indicate the pathological mitoses in the tumor.

In the 3-month dermoscopic follow-up of the scar, the polymorphous vascular pattern consisting of dotted and linear vessels was present. Moreover, in the lower segment of the scar, there was a pinkish structure so-called the “milky red area” (Fig. 2). Because of discordance between the previous diagnosis and the alarming dermoscopic image, the patient underwent subsequent surgical excision of the scar. Based on the clinical and histopathological features a diagnosis of nodular melanoma, epithelioid type, Clark 3, Breslow 2.39-mm was established (Fig. 3). The diagnosis was confirmed by immunohistochemistry-MelanA(+), Ki67 positive in 10% of all malignant cells. Sentinel lymph node biopsy showed metastasis.

## Discussion

The distinction of melanoma even from a clinically typical Spitz nevus is still challenging, mainly because their dermoscopic and even histopathological features can overlap. The lower extremities are the most common anatomic region for both Spitz nevi and Spitzoid melanomas.^1^, ^2^

The studied patient’s young age and negative family history raised the suspicion of Spitz nevus, however, the evolution of the scar, especially after the second excision was highly dubious, because the Spitz nevi have a low recurrence rate, even after incomplete excision.^3^ Moreover, its dermoscopic features, particularly the dotted and linear vessels, greatly increased the likelihood of the lesion being a melanoma. Unfortunately, the dermoscopic image of the primary lesion of the patient in this present study patient is not known because this diagnostic procedure was missed before surgical excision.

The histopathological examination still remains as the gold standard for Spitz nevus diagnosis although in some cases, even with the use of immunohistochemical markers, an entirely safe diagnosis cannot be made.^4^ Although rare, the incidence of pediatric melanomas has been increasing, and most of them arise de novo rather than in preexisting nevi.^4^ Moreover, most of the pediatric melanomas are located at the lower extremities, with delayed diagnosis resulting in thick lesions and more likely with positive sentinel nodes.^5^

This case highlighted that the pink, rapidly growing nodular lesions in a patient older than 12-years of age should be excised but with a mandatory preceding dermoscopy.

## Financial support

None declared.

## Authors’ contributions

Joanna Pogorzelska-Dyrbuś: Approval of the final version of the manuscript; design and planning of the study; drafting and editing of the manuscript; collection, analysis, and interpretation of data; effective participation in research orientation; intellectual participation in the propaedeutic and/or therapeutic conduct of the studied cases; critical review of the literature.

Beata Bergler-Czop: Approval of the final version of the manuscript; design and planning of the study; critical review of the literature; critical review of the manuscript.

Maciej Kajor: Approval of the final version of the manuscript; effective participation in research orientation; intellectual participation in the propaedeutic and/or therapeutic conduct of the studied cases; critical review of the manuscript.

## Conflicts of interest

None declared.
